# Supplementary material for: CD44, γ-H2AX, and p-ATM Expressions in Short-Term Ex Vivo Culture of Tumour Slices Predict the Treatment Response in Patients with Oral Squamous Cell Carcinoma
Source: Int J Mol Sci. 2022 Jan 14;23(2):877. doi: 10.3390/ijms23020877 (PMC8775909; doi:10.3390/ijms23020877)
Supplement: Supplementary file 1 [file ijms-23-00877-s001.zip › ijms-1530313-supplementary.pdf]

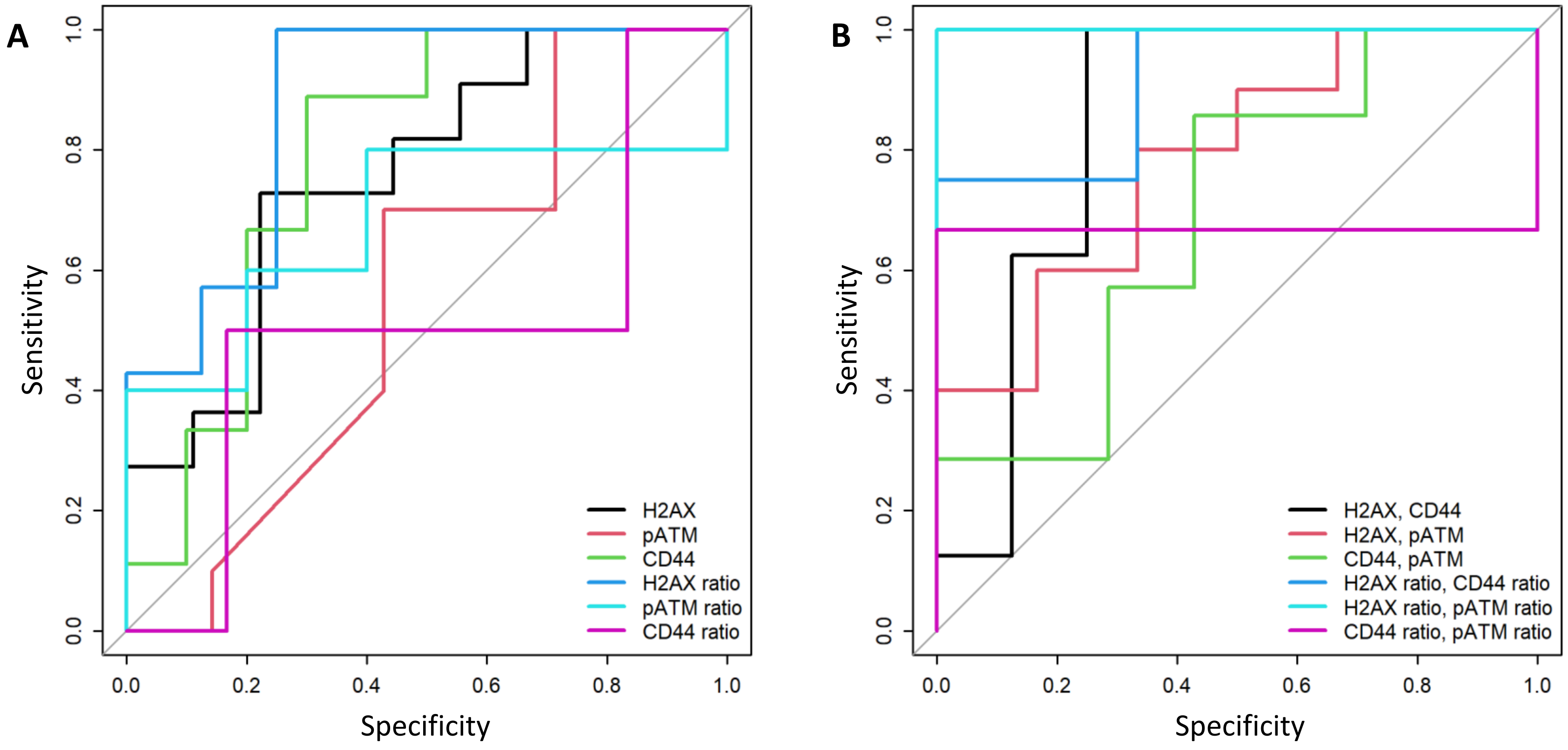

Figure S1 : Receiver operating characteristic (ROC) curve for the different biomarkers (γ-H2AX, pATM, CD44, γ-H2AX 0 Gy/4 Gy ratio, pATM 0 Gy/4 Gy ratio, CD44 0 Gy/4 Gy ratio) alone (A) or combined (B).
